# Cerium-based nanozymes for chemodynamic therapy: tumor microenvironment-responsive mechanisms and applications

**DOI:** 10.3389/fchem.2026.1845768

**Published:** 2026-05-08

**Authors:** Yuan Lu, Yamin Wu, Ke Wen, Zhenzhen Wei, Lu Huang, Mengyao Zhang, Xuetao Huang, Qian Liu

**Affiliations:** Integrated Chinese and Western Medicine Institute for Children Health & Drug Innovation, Jiangxi University of Chinese Medicine, Discipline of Chinese and Western Integrative Medicine, Nanchang, Jiangxi, China

**Keywords:** cerium-based nanozymes, chemodynamic therapy, multi-enzyme activity, tumor, tumor microenvironment

## Abstract

Cerium-based nanozymes (CeNZs) are a class of nanomaterials possessing enzyme-like catalytic activities. Their unique Ce^3+^/Ce^4+^ redox pair and multi-enzyme mimicking activities make them highly advantageous for tumor chemodynamic therapy (CDT). This review systematically summarizes recent research progress in the application of CeNZs for tumor CDT. It focuses on their multi-enzyme catalytic mechanisms and the intelligent regulation of their catalytic behavior by the tumor microenvironment. Furthermore, it elaborates on various CeNZ-based CDT strategies, including single-modality CDT, self-supplying CDT, and combination therapies integrated with photothermal therapy, photodynamic therapy, sonodynamic therapy, and chemotherapy. Finally, the challenges and future directions for the clinical translation of CeNZs are discussed, focusing on precise design, biosafety, and efficacy evaluation. As high-performance and tunable nanocatalytic platforms, CeNZs hold broad prospects for developing efficient and low-toxicity tumor treatment strategies.

## 1 Introduction

Malignant tumor remains one of the major diseases endangering human health and is currently the second leading cause of death worldwide. According to the latest global cancer statistics, there were an estimated 20.0 million new cancer cases and 9.7 million cancer-related deaths in 2022 alone (Bray et al., 2024). Beyond the stark mortality figures, cancer imposes an immense global health burden quantified by disability-adjusted life years (DALYs). The latest data from the Global Burden of Disease Study indicate that cancer accounted for 8.8% (95% uncertainty interval: 7.99–9.67) of total global DALYs in 2021, a metric that encompasses both years of life lost (YLLs) due to premature mortality and years lived with disability (YLDs) (Wu et al., 2024). At present, conventional tumor treatment modalities have significant limitations (Yao et al., 2023; Zhang S. N. et al., 2025), which contribute to persistent high DALY rates, particularly in metastatic settings. Consequently, researchers are actively exploring more precise, targeted, and effective novel therapeutic strategies for tumor treatment (He et al., 2026; Wang H. L. et al., 2025; Zhang Z. Q. et al., 2025) to ultimately reduce this enormous societal burden.

**FIGURE 1:**
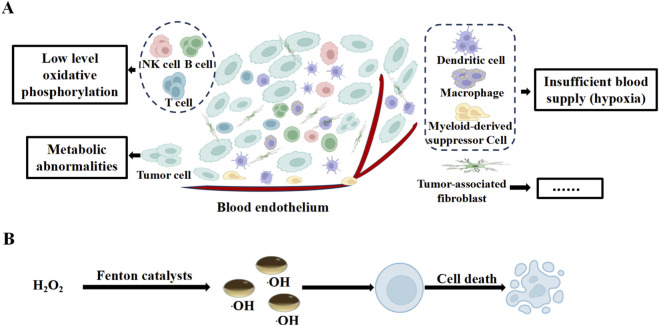
Mechanisms of tumor cell death in the tumor microenvironment. **(A)** Structural features of tumor tissue. **(B)** Mechanisms of tumor cell death.

The tumor microenvironment (TME), which plays a critical role in tumor development, has become a focal point of research. The TME constitutes a complex system composed of diverse cell types and extracellular matrix components (Kang et al., 2016; Cha et al., 2020). Within this system, tumor cells, fibroblasts, and immune cells coexist in a dense network of extracellular matrix (Figure 1A). This intricate physiological architecture establishes a natural physical barrier to drug delivery (Shin and Cheon, 2017). Notably, the rapid proliferation of cancer cells creates a high demand for nutrients, while their reduced oxidative phosphorylation leads to localized lactate accumulation, thereby fostering an acidic microenvironment (Yang et al., 2020; Zhao et al., 2020). Moreover, tumors often exhibit abnormal vasculature and inadequate blood perfusion, resulting in tissue hypoxia (Gao et al., 2010). The accelerated proliferation of malignant cells also disrupts metabolic homeostasis, generating elevated levels of reactive oxygen species (ROS), including hydroxyl radicals (•OH), superoxide anions, and singlet oxygen (Parcero-Bouzas et al., 2024; Wang and Yan, 2021). To counteract the resulting oxidative stress and maintain intracellular redox balance, the levels of reducing molecules such as catalase (CAT), glutathione (GSH), glutathione peroxidase, and superoxide dismutase (SOD) are correspondingly upregulated (Gao et al., 2011; Pan et al., 2018). Additionally, tumor cells frequently overexpress or secrete various enzymes, such as tissue proteases, matrix metalloproteinases, and hyaluronidase, which facilitate tumor growth and metastasis (Hu et al., 2012; Kim et al., 2008). Apart from further promoting tumor progression and metastasis, the TME also significantly attenuates the therapeutic efficacy of diverse clinically available cancer treatment modalities via a series of mechanisms. Therefore, modulating the TME has been recognized as a promising approach for improving cancer treatment (Yan et al., 2025).

Chemodynamic therapy (CDT) is an emerging tumor treatment technology that leverages the unique characteristics of the TME (Sun X. H. et al., 2024; Thirumurugan et al., 2024). Its fundamental principle relies on the higher concentration of hydrogen peroxide (H_2_O_2_) in the TME compared to normal tissues. Transition metal ions catalyze Fenton or Fenton-like reactions, converting H_2_O_2_ into highly cytotoxic •OH (Feng et al., 2023; Jia et al., 2022; Wang et al., 2023). These •OH induce DNA strand breaks, protein dysfunction, and lipid peroxidation of cell membranes, ultimately leading to tumor cell death through apoptosis and necrosis pathways (Han J. Y. et al., 2024; Yu J. et al., 2024) (Figure 1B). Compared with conventional therapies, CDT offers distinct advantages (Table 1). Due to the low H_2_O_2_ level and neutral pH in normal tissues, Fenton or Fenton-like reactions are inefficient, rendering CDT highly selective to tumors with minimal off-target effects (Fang et al., 2019; Gao et al., 2019; Yang et al., 2022). Moreover, CDT does not require external energy input, thereby avoiding limitations such as the limited tissue penetration of light in photodynamic therapy and the dependence on ultrasound equipment and precise targeting in sonodynamic therapy, making it suitable for treating deep-seated tumors (Hou et al., 2024). Nevertheless, CDT still faces several challenges. Firstly, the intratumoral H_2_O_2_ supply is often insufficient to sustain efficient Fenton or Fenton-like reactions (Gupta et al., 2018; He et al., 2024). Secondly, high levels of antioxidants such as GSH in the TME can rapidly scavenge •OH, compromising the therapeutic efficacy (Gao et al., 2022; Han D. et al., 2024; Huang et al., 2022). Additionally, the targeted delivery and controlled release of nanocatalysts require further improvement, and the long-term biosafety of metal ions necessitates systematic evaluation (Fang et al., 2022). Addressing these challenges calls for innovative strategies in material design and nanotechnology (Tang et al., 2019).

**TABLE 1:** Representative experimental performance comparison between CeNZs-based chemodynamic therapy and conventional cancer therapies.

Comparison item	Conventional cancer therapies	Chemodynamic therapy (CDT)	Representative key experimental data of CeNZs
Targeting specificity	Limited; chemotherapy *via* systemic biodistribution; radiotherapy/surgery *via* physical/anatomical localization	TME-responsive activation; theoretically minimal impact on normal tissues	Exhibited >60% inhibition against HeLa, HepG2, A549, 4T1 tumor cells, while maintaining >90% viability in L02 and HUVEC normal cells (Ma et al., 2022)
Systemic toxicity	Chemotherapy: high systemic toxicity; radiotherapy: collateral damage; surgery: trauma/infection risks	Localized catalytic reactions minimize systemic exposure; reduced off-target toxicity	Ceria nanodots showed high cytotoxicity toward HeLa cells (viability ∼25%, pH 6.0) but were nontoxic and protective to MSCs (>90% viability, pH 7.4) (Wang H et al., 2022)
Drug resistance	Chemotherapy: multidrug resistance; radiotherapy: intrinsic/acquired radio resistance	ROS-mediated cell death; mechanistically distinct from conventional resistance pathways	CeO_2_ inhibited MCF-7/ADR cells by 88.2%, while free DOX showed only 12.7% inhibition (Tang et al., 2023)
Therapeutic controllability	Dosing constrained by toxicity (chemotherapy) or normal tissue tolerance (radiotherapy); narrow therapeutic window	Tunable *via* catalyst design, TME modulation, or external stimuli (e.g., light, ultrasound)	Mn–O–Ce nanozyme: 808 nm NIR enhanced POD activity 3.2-fold, raising tumor inhibition in 4T1 mice from 42% to 89% (Ye et al., 2025)
Material design flexibility	Fixed molecular structures; limited synthetic flexibility	Highly tunable *via* composition, structure, and surface functionalization	CeO_2_Mn_1_._08_O_x_ (Mn–Ce) heterojunction: several-fold higher POD activity than pure CeO_2_; ∼90% tumor inhibition in 4T1 mice *via* combined therapy (Qiao et al., 2025)

**FIGURE 2:**
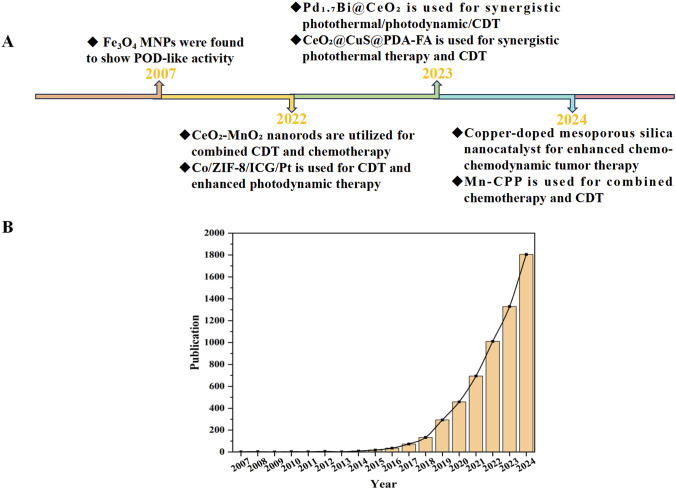
Advances in cerium-based nanozymes. **(A)** Representative advances in nanozymes for cancer therapy in recent years. **(B)** Number of published papers on nanozyme research by the end of 2024. The data is based on the Web of Science.

**FIGURE 3:**
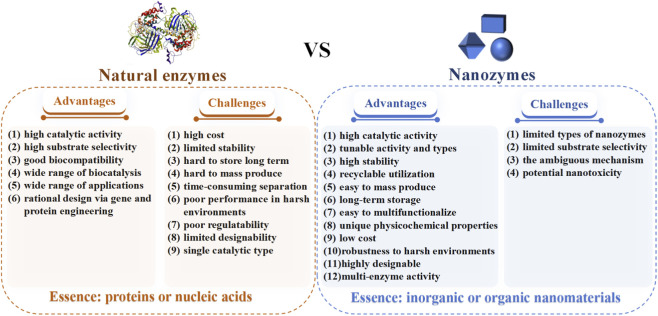
Comparison between natural enzymes and nanozymes.

Nanozymes are a class of novel materials that integrate the unique physicochemical properties of nanomaterials with enzyme-like catalytic activities. They have become a research hotspot since the discovery and characterization of iron oxide (Fe_3_O_4_) nanozymes by Yan’s team in 2007 (Liang and Yan, 2019) (Figure 2). Nanozymes exhibit enzyme-like catalytic activities, enabling them to catalyze the conversion of typical enzyme substrates under physiological conditions while adhering to enzyme kinetic principles (Zhang R. F. et al., 2025). So far, several preparation methods of nanozymes have been reported, including chemical reduction, hydrothermal method, self-assembly method, biomimetic mineralization and sol-gel method. The selection of a suitable preparation method for nanozymes is dictated by their material composition, morphology, activity requirements, and intended application, as different methods yield nanostructures with varying properties. Compared to natural enzymes, nanozymes offer advantages such as high stability, catalytic diversity, and tunability (Figure 3). In addition to these physicochemical merits, a critical benchmarking of catalytic performance is required to validate their functional superiority. For instance, certain CeNZs exhibit peroxidase (POD)-like activity with Michaelis-Menten constant (K_m_) values for H_2_O_2_ in the millimolar to sub-millimolar range (e.g., 0.04–4.0 mM) (Cao et al., 2022; Oliveira et al., 2019), which are comparable to or lower than that of natural horseradish peroxidase (HRP, K_m_≈3.7 mM) (Alshatteri et al., 2023). Such robust catalytic performance underpins the broad application prospects of nanozymes in biomedicine, particularly in CDT for targeted cancer through ROS generation. Additional key applications include antibacterial treatment, photothermal therapy (PTT), photodynamic therapy (PDT), starvation therapy (ST) and biomarker detection. Beyond these established therapeutic and diagnostic functions, there is a growing impetus to integrate nanozymes with emerging nanoscale tools (Marcuello et al., 2025) and numerical machine learning methods (Arravalli et al., 2025) for the early prognosis of cancer diseases. Such integration not only facilitates the ultrasensitive detection of early-stage biomarkers but also enables predictive modeling of therapeutic outcomes, thereby reinforcing the necessity of investing resources in the rational design of smart, stimuli-responsive treatments against tumor malignancies. In CDT, nanozymes could selectively kill tumor cells by catalyzing the Fenton or Fenton-like reaction of endogenous H_2_O_2_ within the TME, generating highly toxic •OH. Currently, nanozymes widely applied in CDT primarily include iron-based, manganese-based, and cobalt-based nanomaterials (Wang Z. R. et al., 2022; Yu H. L. et al., 2024; Zhang et al., 2024). Iron-based nanozymes are constrained by pH limitations, manganese-based nanozymes suffer from substrate competition, and cobalt-based nanozymes face issues of oxygen dependence and biosafety. Collectively, these critical drawbacks severely limit their therapeutic efficacy and hinder their clinical applications.

To address the above limitations of conventional nanozymes in CDT, cerium-based nanozymes (CeNZs) have emerged as highly promising candidates due to their unique advantages. CeO_2_ is a representative cerium-based nanozyme. It not only possesses a high specific surface area and excellent oxygen storage capacity but also exhibits distinctive catalytic behavior different from that of natural enzymes (Tan Z. C. et al., 2022). CeO_2_ nanozymes demonstrate POD-like activity over a broad range of pH and temperature. The presence of mixed valence states (Ce^3+^/Ce^4+^) on their surface enables the material to maintain high catalytic efficiency across diverse environments. Furthermore, CeO_2_ can mimic CAT activity. In physiological or weakly acidic TME conditions, the reversible conversion between Ce^3+^ and Ce^4+^ facilitates the decomposition of endogenous tumor H_2_O_2_ into O_2_ (Qiao et al., 2025). This process not only effectively alleviates tumor hypoxia, counteracting the suppression of nanozyme activity caused by low oxygen levels, but also supplies O_2_ as a substrate for subsequent reactions. The generated O_2_ can further promote the production of ROS, such as •OH, thereby synergistically enhancing the therapeutic efficacy of CDT. Integrating important recent findings from *in vitro* and *in vivo* studies, this review provides a systematic reference for understanding the mechanisms and technological development of CeNZs in CDT, aiming to advance their fundamental research and clinical translation.

## 2 Enzyme-like activities of CeNZs

### 2.1 POD-like activity

POD-like activity of CeNZs is one of their most notable enzyme-like properties, playing a central role in tumor therapy (Zhang et al., 2020). This activity originates intrinsically from the reversible redox cycle between Ce^3+^ and Ce^4+^ coexisting on the nanocrystal surface. Under acidic conditions, Ce^3+^ sites efficiently catalyze the heterolytic cleavage of H_2_O_2_, generating highly toxic •OH while being oxidized to Ce^4+^. Subsequently, the Ce^4+^ sites can oxidize chromogenic substrates such as 3,3′,5,5′-tetramethylbenzidine or intracellular biomolecules, concurrently being reduced back to Ce^3+^, thereby completing an efficient catalytic cycle (Li et al., 2019). This mechanism follows the classical ping-pong model. A key characteristic of this POD-like activity is its pronounced pH dependence, exhibiting maximum activity in acidic environments and lower activity at physiological neutral pH. Liu et al. found that the POD-like activity of Cu-CeO_2_ nanoparticles is optimal in the acidic pH range of 3.5–4.0, but decreases significantly under neutral or slightly acidic conditions (Liu et al., 2024). This property aligns well with the weakly acidic nature of the TME. Although the optimal pH is slightly lower than the actual pH of the TME, the catalytic efficiency within the TME remains significantly higher than that in normal tissues. Grygorova et al. found that ultrasmall CeO_2-x_@β-CD nanoparticles exhibit high pH-dependent POD-like activity toward 3,3′,5,5′-tetramethylbenzidine, thereby ensuring high selectivity for tumor cells, which have a lower pH than normal cells (Grygorova et al., 2025). This inherent pH selectivity enables CeNZs to function as catalytic drugs, being specifically activated at the tumor site to generate high levels of •OH *in situ* for killing cancer cells, while remaining relatively inert in normal tissues. This significantly enhances the selectivity of CDT and reduces systemic side effects.

### 2.2 CAT-like activity

CAT-like activity is another core function of CeNZs, defined as the ability to efficiently catalyze the decomposition of H_2_O_2_ into harmless O_2_ and H_2_O under weakly alkaline conditions. Although the catalytic mechanism still relies on the rapid redox cycling of the surface Ce^3+^/Ce^4+^ pair, its pathway diverges significantly from that of POD-like activity. Specifically, CAT-like activity proceeds via two consecutive electron transfer steps. Firstly, one H_2_O_2_ molecule gains electrons at a Ce^4+^ site, reducing it to Ce^3+^ while generating O_2_. Subsequently, another H_2_O_2_ molecule loses electrons at a Ce^3+^ site, oxidizing it back to Ce^4+^ and yielding two H_2_O molecules, thereby completing the catalytic cycle (Baldim et al., 2018; Wu et al., 2019). This process scavenges accumulated intracellular H_2_O_2_, preventing its conversion into highly toxic •OH via Fenton/Fenton-like reactions and thus playing a crucial role in maintaining cellular redox homeostasis.

Notably, in contrast to the acidic pH optimum of POD-like activity, the CAT-like activity of CeNZs reaches its peak under physiologically neutral conditions. This pH-responsive behavior enables CeNZs to exert antioxidant effects in normal tissues while switching to pro-oxidant mode within the TME. Furthermore, the O_2_ generated from CAT-like decomposition of endogenous H_2_O_2_ holds significant therapeutic value. It alleviates tumor hypoxia, suppressing the expression of hypoxia-inducible factor HIF-1α and its downstream pathways. This reversal of hypoxia mitigates associated therapeutic resistance, including chemotherapy resistance, immunosuppression, and resistance to radiotherapy and PDT (Kim et al., 2019). For instance, the mesoporous silica/nano-cerium oxide composite (MSN-Ce@SP/PEG) exhibits both POD-like activity and CAT-like activities. Leveraging its prominent CAT-like activity, it efficiently decomposes tumoral H_2_O_2_ into O_2_ to relieve hypoxia, while its pronounced POD-like activity elevates intracellular ROS levels. This synergistic mechanism ultimately suppresses tumor recurrence and metastasis (Wang Y. H. et al., 2025). Therefore, utilizing the CAT-like activity of CeNZs to modulate tumor oxygen metabolism provides a key strategy for developing novel synergistic cancer therapies.

### 2.3 Other enzyme-like activities

Beyond POD-like and CAT-like activities, CeNZs exhibit other significant enzymatic functions, including SOD-like and oxidase (OXD)-like activities. These multifunctional capabilities collectively form the foundation for their applications in biocatalysis and antioxidant therapy (Jiang et al., 2026; Liu et al., 2022). The SOD-like activity demonstrated by CeNZs enables efficient dismutation of superoxide anion radicals (O_2_•^-^) into H_2_O_2_ and O_2_ (Han X. et al., 2024; Peng et al., 2025). This mechanism plays a critical role in intracellular antioxidant defense by effectively interrupting free radical chain reactions and mitigating oxidative stress-induced damage to biomacromolecules and tissue dysfunction. Furthermore, CeNZs exhibit OXD-like activity. This activity enables them to catalyze the oxidation of various substrates using O_2_ as the electron acceptor, without the need for exogenous H_2_O_2_ (Sun Q. J. et al., 2024). This catalytic process primarily relies on the surface Ce^4+^ sites acting as strong oxidants and Lewis acid centers, facilitating oxidation by directly extracting electrons from substrate molecules. This characteristic makes CeNZs particularly promising for biosensing and *in vitro* diagnostics, especially in analytical systems sensitive to or requiring avoidance of H_2_O_2_ interference. Additionally, Singh et al. reported that CeVO_4_ nanozymes exhibit cytochrome coxidase (CcO)-like activity under physiological pH conditions. This activity involves electron transfer among Ce^3+^, Ce^4+^, and V^5+^ ions, enabling the reduction of oxygen to water. Notably, the Ce^3+^/Ce^4+^ ratio in CeVO_4_ plays a regulatory role in its CcO-like activity (Singh and Mugesh, 2019).

Although the POD-like activity of CeNZs serves as the primary driver of CDT by generating lethal hydroxyl radicals, the aforementioned auxiliary activities—SOD, OXD, and CcO—can be strategically harnessed to overcome the intrinsic limitations of CDT and amplify therapeutic efficacy. The SOD-like activity of CeNZs provides a self-sustaining substrate feed for the Fenton-like catalytic cycle. Given that mitochondrial dysfunction in cancer cells frequently leads to O_2_•^-^ overproduction, SOD-mimetic CeNZs convert endogenous O_2_•^-^ into H_2_O_2_, thereby effectively elevating the local concentration of the POD substrate within the TME. This not only augments the yield of cytotoxic •OH but also circumvents the reliance on the limited endogenous H_2_O_2_ pool, enabling sustained CDT even under conditions of moderate oxidative stress. Concurrently, the OXD-like activity of CeNZs enables direct generation of ROS from molecular oxygen, bypassing the absolute requirement for H_2_O_2_ (Meng et al., 2024). This is particularly advantageous in hypoxic tumor regions, where H_2_O_2_ levels may be insufficient to drive robust POD catalysis. By leveraging OXD-mimetic activity, CeNZs can initiate oxidative damage to lipids, proteins, and DNA via superoxide or hydrogen peroxide intermediates, thereby complementing •OH-mediated cytotoxicity and extending the therapeutic window to oxygen-deprived tumor niches. Furthermore, the CcO-like activity of CeNZs offers a unique avenue for modulating mitochondrial respiration. CcO-mimetic CeNZs, by facilitating electron transfer to oxygen, can potentially restore mitochondrial membrane potential or, conversely, disrupt electron transport chain homeostasis, leading to amplified ROS leakage and enhanced susceptibility of tumor cells to CDT-induced apoptosis (Yang et al., 2025). Collectively, the rational integration of SOD-, OXD-, and CcO-like activities with the core POD-driven CDT mechanism transforms CeNZs from simple Fenton catalysts into multifunctional nanomedicines capable of orchestrating a multi-pronged oxidative assault on tumors while mitigating the constraints imposed by the heterogeneous TME. This strategic synergy holds considerable promise for improving clinical outcomes in cancer therapy.

However, it is noteworthy that CeNZs also possess intrinsic ROS scavenging properties due to their CAT-like, SOD-like, and antioxidant activities. Excessive ROS scavenging may neutralize cytotoxic •OH generated by POD-like activity, thereby compromising the antitumor efficacy of CDT. To address this critical contradiction, rational structural design must be employed to selectively amplify pro-oxidant activity for CDT and spatially or temporally suppress ROS scavenging effects. Strategies including valence state engineering to increase surface Ce^3+^ ratio, TME-responsive surface masking, heterostructure construction with Fenton-active metals, and spatial functional partitioning can effectively prevent the antioxidant behavior of CeNZs from weakening CDT performance. These design principles are essential to guide the development of CeNZs with enhanced pro-oxidant specificity and improved therapeutic outcomes, providing important insights for future research.

## 3 Structure-activity relationships and regulatory mechanisms of CeNZs

**FIGURE 4:**
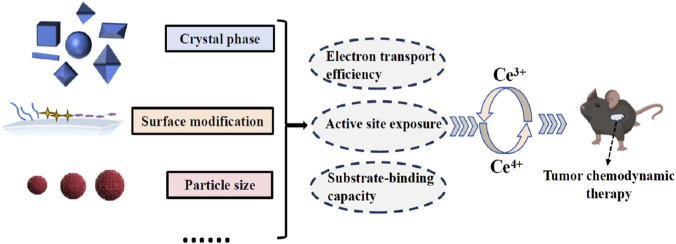
Key factors influencing the catalytic activity of cerium-based nanozymes in tumor chemodynamic therapy.

The multi-enzyme catalytic activities of CeNZs are dictated by three key structural factors, including crystal phase, particle size, and surface modification (Figure 4). These factors collectively govern the reversible Ce^3+^/Ce^4+^ redox cycle, the central determinant of catalytic performance in CDT, by modulating electron transport efficiency, active site exposure, and substrate-binding capacity.

Among the various crystal phases, cubic fluorite CeO_2_ exhibits optimal multi-enzyme activity owing to its high lattice regularity and abundant oxygen vacancies (Table 2), which facilitate the Ce^3+^/Ce^4+^ redox cycling (Cai et al., 2025). In contrast, hexagonal or amorphous CeNZs show greater lattice disorder and unstable active site coordination, leading to the decreased catalytic activity. Shi et al. synthesized a series of CeO_2_ nanoparticles with rod-like, octahedral, and cubic morphologies, and found that morphology variations shift the position of oxygen vacancy-dependent surface states within the band structure, thereby modulating enzymatic activities (Shi et al., 2023). It has been proven that the structural morphology of nanozymes has a significant impact on their activity. Besides, size effect plays another critical role. CeNZs with diameters ≤5 nm generally possess larger specific surface areas and higher densities of surface active sites, resulting in two-to three-fold higher catalytic activity compared with large-sized particles (Zhou et al., 2025). Filippova et al. synthesized CeO_2_ nanoparticles with sizes of 2.5, 2.8, 3.9, and 5.1 nm, proving that particles with smaller sizes exhibit higher catalytic activities under identical pH conditions (Filippova et al., 2023). Surface modification offers a versatile platform for tuning the enzymatic activities of CeNZs through distinct mechanisms. For instance, inert coatings such as polyethylene glycol improve the dispersion of CeNZs without compromising accessibility of active sites (Xu et al., 2024). Noble metal doping or metal oxides compositing optimizes the electronic structure, thereby accelerating the Ce^3+^/Ce^4+^ redox cycling (Zhong et al., 2024). Additionally, positively charged modifiers enhance the affinity toward negatively charged substrates, whereas strategies based on ligand-to-metal charge transfer confer photo-responsive catalytic properties.

**TABLE 2:** Effects of morphology and crystal structure on the CDT performance of cerium-based nanozymes (CeNZs).

Types of CeNZs	Morphology	Dominant exposed facets	Surface Ce characteristics	Dominant enzyme-like activities	Key features
CeO_2_	Octahedron (Tan Z. C. et al., 2022)	{111}	Acidic Ce species; lowest electron density	Phosphatase, SOD	POD activity lower than cube/rod
CeO_2_	Cube (Shi et al., 2023)	{100}	Electron-rich Ce species	SOD, POD, CAT, OXD, Haloperoxidase	Inert for phosphatase; optimal for substrate oxidation with H_2_O_2_
CeO_2_	Rod (Cheng et al., 2024)	{110}, {100}	High oxygen vacancy content	CAT, POD, Haloperoxidase	Lateral {110} facets responsible for POD; tip {111} facets for Haloperoxidase
CeO_2_	Sphere (Akdogan et al., 2022)	{111}	Balanced oxygen vacancies; moderate Ce^3+^/Ce^4+^ ratio	SOD, CAT, POD	Suitable for broad-spectrum antioxidant applications
CeO_2_	Tube (Cao et al., 2021)	{110}, {100}	Ultrahigh oxygen vacancy density; elevated Ce^3+^/Ce^4+^ ratio	POD, CAT, SOD, Haloperoxidase	Dual active surfaces (inner/outer)

Despite these versatile regulatory strategies, CeNZs face several unique hurdles compared with traditional iron-based nanozymes, which impose additional constraints on their rational design for CDT. The intrinsic Ce^3+^/Ce^4+^ redox cycle of CeNZs is inherently sluggish. The low conversion rate between Ce^3+^ and Ce^4+^ limits the overall catalytic turnover, often necessitating extrinsic doping—such as Mn incorporation—to introduce donor energy levels and accelerate electron transfer (Shuai et al., 2024). In contrast, the POD-like activity of iron oxide nanozymes under acidic conditions predominantly originates from a surface-mediated mechanism that directly generates complexed hydroxyl radicals and high-valent iron-oxo species, without relying on the classical Fenton chemistry pathway (Wan et al., 2022). Furthermore, the surface chemistry of CeNZs is considerably more complex than that of iron oxides, with multiple enzyme-like activities coexisting on their surfaces. While this multi-enzyme nature confers multifunctionality, it also introduces a pronounced specificity challenge. Achieving precise control over a single desired reaction pathway typically necessitates facet-selective engineering to decouple competing H_2_O_2_ activation pathways. Consequently, the catalytic performance of CeNZs is intricately linked to their crystal phase, size, and surface modification, which collectively govern Ce^3+^/Ce^4+^ redox dynamics and substrate interactions. A rational design of these CeNZs structural parameters, coupled with strategies to mitigate the aforementioned regulatory hurdles, enables the development of TME-responsive nanozymes with enhanced catalytic activity and tumor targeting capability, offering a promising strategy to improve CDT efficacy.

## 4 Regulatory mechanism of the TME on the catalytic activity of CeNZs

The TME is a critical factor modulating the catalytic behavior of CeNZs through its characteristic features: acidic pH, high GSH levels, and hypoxia. These factors precisely regulate the types and efficiency of CeNZs enzyme-like activities in the tumor region by directly or indirectly influencing the valence state equilibrium of the cerium active centers and the associated electron transfer processes. A thorough understanding of this multi-dimensional regulation is crucial for the rational design of intelligent, TME-responsive nanocatalytic agents.

### 4.1 pH responsiveness

The catalytic behavior of CeNZs is highly sensitive to pH, primarily because variations in pH significantly alter the Ce^3+^/Ce^4+^ ratio, which in turn dictates the type and efficiency of enzyme-mimicking activities. Under acidic TME conditions, CeNZs predominantly exhibit POD-like and OXD-like activities, whereas neutral pH favors OXD-like activity. Liu et al. demonstrated this pH-dependent behavior using a hexanuclear Ce-MOF cluster, which showed strong OXD-like activity at neutral pH (Liu et al., 2025). The enhanced POD-like activity under acidic conditions has been exploited for ROS-mediated tumor therapy. Additionally, Ali et al. synthesized CeZrO_4_ nanoparticles via a green method. These nanoparticles exhibited dual POD-like and OXD-like activities with optimal catalytic performance at pH 4, correlating with high ROS generation capacity in acidic media (Ali et al., 2022). Building upon this pH-responsive valence-switching property of CeNZs, researchers have developed advanced therapeutic platforms. One example is a self-regulating nano-cerium-doped polymer (cyclopentadithiophene-benzo-thiadiazole, SPN-C23), which was regarded as an ROS converter upon near-infrared (NIR) laser irradiation in the acidic TME, catalyzing the conversion of superoxide to H_2_O_2_ and enhancing tumor cell killing (Zhu et al., 2017). Furthermore, Gong et al. developed a two-dimensional Ce-based nanozyme (He@Ce-BTB nMOL) that integrates CAT-, POD-, and glutathione oxidase-like activities. This nanozyme is specifically activated within the acidic TME, where it promotes lipid peroxidation and induces ferroptosis, achieving selective elimination of tumor cells with minimal effects on normal tissues. The significant therapeutic efficacy demonstrated in both *in vitro* and *in vivo* studies underscores the promise of CeNZs in catalytic tumor therapy (Gong et al., 2025). Therefore, pH responsiveness endows CeNZs with the capacity to dynamically adjust their catalytic behavior according to the local microenvironment, allowing for precise and efficient ROS regulation within the TME.

### 4.2 GSH responsiveness

GSH, a key intracellular reducing agent, exerts a concentration-dependent dual regulatory effect on the catalytic activity of CeNZs. The underlying mechanism involves dynamic electron exchange between GSH and the Ce^3+^/Ce^4+^ redox couple on the CeNZs surface. At relatively low concentrations, GSH acts as a beneficial reducing cofactor that maintains the catalytic cycle of CeNZs. Specifically, in the POD-like reaction, after Ce^4+^ catalyzes H_2_O_2_ conversion to •OH and is reduced to Ce^3+^, GSH provides reducing equivalents to regenerate Ce^4+^, restoring the catalytic capacity of the active centers and promoting sustained ROS generation. This mechanism is particularly critical in H_2_O_2_-limited microenvironments. However, GSH exerts an inhibitory effect on CeNZs catalysis in the abnormally high GSH environment of tumor cells (Wang et al., 2018).

To overcome this limitation, researchers have developed strategies to deplete local GSH, including the introduction of manganese ions or chemotherapeutic agents. Zeng et al. loaded cisplatin into polydopamine-coated bimetallic cerium-manganese metal-organic framework (Ce-Mn MOF) nanoparticles (Zeng et al., 2022). Under microwave irradiation, these nanoparticles catalyze ROS decomposition into O_2_ to alleviate tumor hypoxia while reducing intracellular GSH levels via redox reactions. The remodeled TME not only enhanced immunogenic cell death induced by cisplatin and microwave thermal therapy but also promoted the polarization of pro-tumor M2 macrophages towards the anti-tumor M1 phenotype. Similarly, the CeO_2_Mn1.08Ox nanozyme, prepared via an H_2_SO_4_/KMnO_4_ oxidation method, exhibits multi-enzyme mimetic activities under acidic conditions and achieves efficient TME modulation by alleviating tumor hypoxia and depleting GSH. These examples illustrate that integrating GSH-depleting capabilities into CeNZ-based platforms represents a promising strategy to counteract the inhibitory effects of the TME and enhance oxidative stress-mediated cytotoxicity.

### 4.3 Hypoxic responsiveness

Hypoxia, a hallmark of solid tumors, arises from the imbalance between inadequate oxygen supply due to abnormal tumor vasculature and increased oxygen consumption by rapidly proliferating tumor cells (Vaupel and Mayer, 2007). This hypoxic state not only impairs the efficacy of conventional cancer therapies but also plays a crucial role in regulating the catalytic activity of CeNZs, particularly in CDT. The core of this regulatory mechanism involves the intrinsic CAT-like activity of CeNZs, which catalyzes the decomposition of endogenous H_2_O_2_ into O_2_, thereby alleviating local hypoxia. The hypoxia amelioration creates a more favorable catalytic microenvironment for the oxygen-dependent enzymatic reactions and activates the OXD-like activity of CeNZs. This activation promotes the conversion of O_2_ into highly toxic O_2_•^-^, establishing a self-enhancing catalytic cycle that amplifies the tumor-killing effect. Several studies have exploited this mechanism to develop effective therapeutic platforms.

For instance, cerium single-atom nanozymes, prepared by immobilizing Ce single atoms on ZIF-8 carrier, efficiently decompose H_2_O_2_ in the TME to generate O_2_ and relieve hypoxia via their CAT-like activity. Subsequently, the increased O_2_ concentration further activated their OXD-like activity to catalyze the conversion of O_2_ to O_2_•^-^, thereby enhancing the tumor-killing effect. Similarly, Wang et al. developed a nanozyme composite composed of mesoporous silica and nano-ceria (MSN-Ce@SP/PEG). This nanozyme exhibits CAT-like activity to convert H_2_O_2_ into O_2_ for hypoxia alleviation, while the improved oxygenation simultaneously enhances its POD-like activity to promote •OH generation. Therefore, it has been proven that harnessing the hypoxia-alleviating capability of CeNZs not only enhances CDT efficacy but also provides a strategic approach to overcome resistance in multimodal cancer therapy.

## 5 Application of CeNZs in CDT of tumors

CDT is a promising tumor treatment strategy that relies on TME-specific catalytic reactions to achieve selective therapy. CeNZs offer distinct advantages in CDT owing to their reversible Ce^3+^/Ce^4+^ redox pairs, multiple enzyme-mimicking activities, and pH-responsive properties. Recent advances have expanded the application of CeNZs from single-modality CDT to synergistic strategies that combine multiple therapeutic approaches (Table 3). These synergistic systems enhance the generation of •OH through external energy input or self-supplied substrates and amplify oxidative stress in tumor cells *via* multi-mechanistic cooperation, thereby addressing the limitations of standalone CDT. This section elaborates on the design concepts and mechanisms of CeNZs in single-modality CDT, self-supplying CDT, and synergistically enhanced CDT.

**TABLE 3:** Comparison of several different types of CDT.

Types	Single-modality CDT	Self-supplying CDT	Synergistically enhanced CDT
Mechanism	Fenton/Fenton-like reaction using endogenous H_2_O_2_ to generate •OH.	Integrates H_2_O_2_-generation module (e.g., metal peroxides, GOx) to supply substrate *in situ*	Combines CDT with other modalities (e.g., photothermal, photodynamic, chemotherapy)
Key feature	TME-dependent; no external energy input	Overcomes insufficient endogenous H_2_O_2_; enhances •OH yield	Synergistic effect between CDT and complementary therapy
Advantage	Simple design; high specificity; minimal systemic toxicity	Addresses substrate limitation; significantly improved therapeutic efficacy	Superior efficacy compared to monotherapy; potential to overcome tumor resistance
Limitation	Constrained by low TME H_2_O_2_ levels and catalytic efficiency	More complex material design; requires precise spatiotemporal control	Increased system complexity; potential for additional side effects
Representative materials	Hollow mesoporous Mn/Zr co-doped CeO _2_ tandem nanozyme ( Dong et al., 2022 ); CeO_2_/GNRs-PEG ( Lin et al., 2025 ); porous CeO_2_ nanorods ( Tian et al., 2020 )	CeO_2_ QD-modified MgO_2_ nanosheets (Yan et al., 2023); Pt/CeO_2_-PEG-GOx (Ding et al., 2025); TCP-PDA-CaO_2_-CeO_2_(Wang et al., 2024)	HCeO_2_@ICG-RGD (Tan H. X. et al., 2022); CeO_2_@CuS@PDA-FA (Zhang et al., 2023); Au@CeO_2_-Pd-PFH(Sheng et al., 2023); DOX@Cu-CeO_2_(Cheng et al., 2021); DOX@PCMNR-RGD (Chen et al., 2022)

### 5.1 Single-modality CDT

Single-modality CDT leverages endogenous TME characteristics, utilizing transition metal-based nanozymes to convert endogenous H_2_O_2_ into •OH via Fenton/Fenton-like reactions without external energy input. CeNZs effectively catalyze Fenton-like reactions through their surface Ce^3+^ sites under weakly acidic conditions to generate •OH. The resulting Ce^4+^ can be self-reduced back to Ce^3+^ by the overexpressed GSH in tumor cells, establishing a persistent catalytic cycle while disrupting the tumor redox homeostasis. Moreover, the pH responsiveness of CeNZs enables POD-like activity in the acidic TME and protective CAT-like activity under neutral conditions in healthy tissues. Several studies have optimized this strategy. Dong et al. developed a hollow mesoporous manganese/zirconium (Mn/Zr) co-doped CeO_2_ tandem nanozyme, which enhances CDT by boosting SOD-like and POD-like activities while suppressing CAT-like activity (Dong et al., 2022). Lin et al. reported a stable CeO_2_/GNRs-PEG nanocomposite with multiple enzyme-mimicking activities, which not only catalyzes the conversion of H_2_O_2_ to •OH but also decomposes H_2_O_2_ to O_2_ (Lin et al., 2025). Then, the O_2_ is further converted into O_2_•^-^, intensifying oxidative stress in the TME and inducing tumor cell death. Additionally, Tian et al. constructed a pH-responsive composite of porous CeO_2_ nanorods and polystyrene sulfonate that exhibits high oxidative activity to generate O_2_•^-^ for CDT in the acidic TME, while showing minimal activity under neutral condition, thereby reducing damage to healthy tissues (Tian et al., 2020). The composite forms localized nanoreactors via its large hydrodynamic diameter, facilitating charge transfer between the catalyst and substrates and enhancing the oxidative reactions. The relevant *in vitro* and *in vivo* results confirm this composite as a promising candidate for selective tumor CDT.

### 5.2 Self-supplying CDT

To address the insufficient endogenous H_2_O_2_ in TME for single-modality CDT, self-supplying CDT strategies have been developed. For example, a novel CDT agent based on CeO_2_ quantum dot-decorated MgO_2_ nanosheet was designed (Yan et al., 2023). Under acidic TME conditions (pH 6.5), MgO_2_ nanosheets hydrolyze to generate substantial H_2_O_2_, which is subsequently converted into •OH by CeO_2_ quantum dots. The combination of self-supplied H_2_O_2_ and pH-responsive activation significantly enhances the Fenton-like reaction efficiency, achieving effective *in vivo* tumor growth inhibition. Further strategies incorporate additional components to amplify H_2_O_2_ supply. Ding et al. (2025) incorporated platinum (Pt) into CeO_2_ via strong metal-support interactions, forming a Pt/CeO_2_ composite that increased the Ce^3+^/Ce^4+^ ratio and accelerated the electron transfer, boosting the Fenton-like reaction cycling efficiency by 11-fold. Glucose oxidase (GOx), which catalyzes the conversion of glucose to H_2_O_2_ and gluconic acid, was integrated with polyethylene glycol (PEG)-modified Pt/CeO_2_ to form a Pt/CeO_2_-PEG-GOx nanozyme. This design elevates H_2_O_2_ levels and enhances TME acidity, thereby amplifying the Fenton-like reaction. Additionally, Wang et al. (2024) constructed a H_2_O_2_ self-supplying TCP-PDA-CaO_2_-CeO_2_ platform by depositing nanoparticles onto a polydopamine (PDA)-modified 3D-printed tricalcium phosphate (TCP) scaffold. The loaded CaO_2_ nanoparticles release Ca^2+^ and abundant H_2_O_2_ in the acidic TME, which is catalyzed by CeO_2_ nanoenzymes to generate •OH, forming a cascade of H_2_O_2_ self-supply and •OH generation for antitumor effects.

### 5.3 Synergistically enhanced CDT

The limited efficacy of single-modality CDT arises from insufficient endogenous H_2_O_2_ and low intracellular Fenton-like reaction rates. Combining CDT with other therapeutic modalities has therefore become a common strategy to enhance antitumor effects. CeNZs, with their tunable Ce^3+^/Ce^4+^ redox pairs and multiple enzyme-mimicking activities, can enhance •OH generation through energy level modulation, electron-hole separation, and optimization of catalytic centers when integrated with these modalities. They also initiate cascade reactions to achieve self-supplied H_2_O_2_, hypoxia alleviation, and GSH depletion, converging into a synergistic platform with precise targeting and multi-stimuli responsiveness for efficient TME-specific therapy.

PTT is a treatment method that can synergize with CDT. Certain Ce-based nanomaterials exhibit photothermal conversion properties under NIR light irradiation, and the resulting temperature elevation accelerates CDT reaction kinetics. Tan et al. synthesized a hollow pH-sensitive CeO_2_ nanozyme, which was subsequently surface-loaded and modified to obtain HCeO_2_@ICG-RGD, enabling precise tumor targeting (Tan H. X. et al., 2022). The results showed that PTT-synergized CDT induces substantial cytoplasmic ROS generation, leading to efficient tumor cell killing. Similarly, Zhang et al. developed a multifunctional CeO_2_@CuS@PDA-FA nanocomposite, where localized hyperthermia from PTT enhances CDT efficiency, achieving a synergistic PTT/CDT effect with improved therapeutic outcomes (Zhang et al., 2023).

Beyond PTT, PDT has also been widely combined with CDT for cancer treatment. Chen et al. reported a MnO_2_-doped CeO_2_ nanozymes-based nanoagent (Ce6@CMNRs) that enables tumor-specific synchronized activation of CDT and PDT (Chen Y. et al., 2023). Tumor-overexpressed H_2_O_2_ competitively displaces Ce6 molecules from the nanoagent surface, followed by the decomposition to generate •OH for CDT under acidic conditions. Simultaneously, the displaced Ce6 triggers the previously suppressed PDT upon laser irradiation, allowing H_2_O_2_ to activate both CDT and PDT at comparable levels. Furthermore, PDT-induced oxygen deprivation stimulates additional H_2_O_2_ generation, which continuously displaces the residual coordinated Ce6 to fully activate both therapies. Gao et al. designed a nanocomposite (mUCC) that induces apoptosis and ferroptosis via PDT-CDT synergy (Gao et al., 2023). Under NIR irradiation, the electron-hole pairs separation on the CeO_2_/CuO heterojunction triggers a photocatalytic reaction to enhance ROS production, while the nanocomposite acts as a Fenton-like reagent to generate highly toxic •OH, achieving a synergistic apoptosis-ferroptosis effect.

SDT offers an alternative energy input for synergistic CDT. The Au@CeO_2_-Pd-PFH (ACPP) nanocomposite exhibits enhanced sonosensitizing effects and Fenton-like catalytic activity, with exceptional ROS generation efficiency under ultrasound irradiation attributed to its narrow bandgap (1.72 eV) and efficient electron-hole (e^−^-h^+^) separation. Furthermore, ACPP utilizes its POD-like activity to convert endogenous H_2_O_2_ into •OH for CDT and its CAT-like activity to produce O_2_, alleviating hypoxia and enhancing SDT (Sheng et al., 2023). Meanwhile, Xu et al. fabricated PEG-PLGA-coated CeO_2_ nanoparticles loaded with the sonosensitizer chlorin e6, which similarly decomposes H_2_O_2_ into O_2_ to improve O_2_-dependent SDT outcomes and exert POD-like activity for CDT under weakly acidic conditions (Xu et al., 2025).

Chemotherapy has also been integrated with CDT to address the challenges such as drug resistance, poor targeting, and systemic toxicity. Cheng et al. designed a complex using copper-doped CeO_2_ nanoparticles (Cu-CeO_2_ NPs) loaded with doxorubicin (DOX) as the core and cancer cell membranes as the shell (Cheng et al., 2021). Cu doping enhanced POD-mimetic activity of CeO_2_ NPs in the TME, while the cancer cell membrane conferred homotypic targeting. Combined with DOX, this system achieved selective and nearly complete tumor suppression. Under physiological conditions, Cu-CeO_2_ NPs acted as radical scavengers, protecting normal cells from oxidative damage induced by DOX and •OH radicals generated via Fenton-like reactions. Similarly, Chen et al. prepared a nanodrug by coating CeO_2_-MnO_2_ nanoparticles with RGD and DOX (Chen et al., 2022). Under weakly acidic conditions, the nanodrug catalyzes H_2_O_2_ to generate •OH for CDT while triggering DOX release, forming a pH responsive CDT-chemotherapy platform.

Building upon these binary combinations, the rational integration of multiple modalities enables the construction of TME-responsive intelligent nanotheranostic systems that address the limitations of monotherapies through cascade reactions. Yan et al. fabricated a cancer cell membrane-camouflaged, ultrasmall CeO_2_-decorated MnO_2_ composite (mMC) for CDT/PDT/PTT synergy (Yan et al., 2021). CeO_2_ converts endogenous H_2_O_2_ into toxic ROS, while MnO_2_ exhibits NIR-induced photothermal effects and ROS generation for simultaneous PTT and PDT. Besides, MnO_2_ also generates oxygen to alleviate hypoxia and consumes GSH to weaken tumor antioxidant defenses with the thermal effect further boosting the catalytic activity of CeO_2_. Consequently, these multiple synergistic effects achieve markedly enhanced therapeutic efficacy. A core-shell Pd_1_._7_Bi@CeO_2_-ICG (PBCI) nanocomposite similarly achieves multimodal synergy, where both the Pd_1_._7_Bi and CeO_2_ components exhibit dual enzyme-like activities to alleviate tumor hypoxia and enhance PDT, while NIR irradiation amplifies the catalytic activity via photothermal effects. Furthermore, PBCI serves as a NIR fluorescent imaging contrast agent for determining the optimal laser treatment time window *in vivo*. Through these synergistic functions, highly efficient tumor elimination was ultimately achieved (Chen X. Y. et al., 2023). For instance, Mei et al. developed an IP6-coated CeO_2_@Au@IP6 (CeAIP) nanocatalyst, in which the POD-like activity of CeO_2_ generates toxic •OH and alleviates tumor hypoxia, while Au NPs enable PTT via surface plasmon resonance, achieving PTT/ST/CDT triple synergy for colon cancer treatment (Mei et al., 2025). This integrated nanozyme strategy offers novel insights for early diagnosis and treatment of colon cancer, potentially improving cure rates. Akdogan et al. further integrated four modalities by adsorbing dihydroporphyrin e6 onto hollow mesoporous CeO_2_ nanoparticles, coating with PDA, and incorporating GOx (Sungu Akdogan et al., 2024). Under NIR irradiation, this system exhibits reversible photothermal conversion, and the GOx-H-CeO_2_ cascade enhances PDT through ROS generation and hypoxia alleviation, while glucose-mediated •OH generation induces CDT. Without anticancer drugs, this quadruple synergy reduced T98G cell viability to 10.6% *in vitro*.

## 6 Conclusion and perspectives

This review summarizes the unique advantages, catalytic mechanisms, and application strategies of CeNZs in tumor CDT. Leveraging the reversible Ce^3+^/Ce^4+^ redox cycle and multi-enzyme-mimetic activity, CeNZs exhibit TME-responsive behavior that enables •OH generation via POD-like activity, hypoxia alleviation via CAT-like activity, and glutathione depletion to disrupt redox homeostasis. In terms of applications, CeNZs have been utilized not only in single-modality CDT but also in self-supplying CDT systems and multimodal synergistic strategies combining with PTT, PDT, SDT, and chemotherapy.

Despite the considerable potential, the clinical translation of CeNZs still faces several critical challenges. First, the complex physiological environment may compromise the catalytic efficiency and targeting specificity. Second, the biosafety profile of CeNZs requires systematic evaluation, including long-term retention, metabolic pathways, and potential immunogenicity. Third, current therapeutic systems lack sufficient capability for real-time monitoring and precise regulation. Finally, the molecular mechanisms underlying the interactions between CeNZs and the TME remain incompletely understood. Moreover, the synergistic mechanisms, optimization strategies, and existing bottlenecks associated with the combined application of CeNZs-mediated CDT and immunotherapy have yet to be fully elucidated and summarized, which represents a major factor constraining the clinical translation of this field.

Building upon the above-mentioned challenges, and with the aim of advancing this field from fundamental research toward clinical application in an orderly manner, we further distill several concrete action lines that warrant urgent pursuit. First, to address the core issues of diminished catalytic efficiency and insufficient targeting specificity of CeNZs within complex physiological environments, priority should be accorded in the near term to atomic-level precision engineering. This entails the stepwise implementation of interface engineering strategies, including rare-earth element single-atom doping, interfacial functional group modification, and the precise construction of core–shell architectures, in order to establish a structure–activity relationship database that links the electronic structure and surface properties of CeNZs with their enzyme-mimetic catalytic performance. Such efforts will facilitate the identification of optimized formulations exhibiting robust *in vivo* stability and high TME responsiveness. In the medium term, multi-target modification strategies should be pursued, integrating tumor cell-targeting peptides and microenvironment-responsive targeting moieties to achieve specific accumulation and precise localization of the materials at tumor sites, thereby mitigating off-target effects in normal tissues and consolidating the material foundation for efficient *in vivo* catalysis. Collectively, these approaches will provide a stable and efficient delivery and catalytic foundation for the combined application of CDT and immunotherapy, ensuring the synergistic efficacy of the combination therapy.

Second, in response to the fragmented nature of current CeNZs biosafety research and the absence of systematic evaluation frameworks, a standardized biosafety assessment pipeline should be established. This pipeline would encompass tiered investigations spanning *in vitro* cytotoxicity, short term *in vivo* acute toxicity, and long term *in vivo* metabolic toxicity, with comprehensive tracking of material retention pathways, metabolic excretion routes, and potential immunogenicity and genotoxicity. Concurrently, the development of degradable CeNZs should be advanced by prioritizing the integration of cerium based components with carrier scaffolds that exhibit excellent biocompatibility and renal clearability, thereby constructing novel nanozyme systems characterized by degradability and minimal residual burden. Parallel safety validation should be conducted across multiple species and dose regimens to generate comprehensive biosafety dossiers that satisfy the stringent prerequisites of preclinical investigation, with particular emphasis on evaluating the immunogenicity of CeNZs in the context of combined CDT and immunotherapy, so as to avoid immune disorders triggered by the material itself that could compromise the therapeutic efficacy of the combination, while also clarifying the modulatory effects of CeNZs on the tumor immune microenvironment and their potential risks.

Third, to overcome the limitations of current systems in real time monitoring and precise regulation, CeNZs based theranostic platforms should be constructed by integrating multimodal imaging modules, such as those encompassing magnetic resonance, fluorescence, and photoacoustic modalities. Such platforms would enable precise tumor imaging, real time monitoring of drug accumulation, and dynamic feedback on therapeutic efficacy. In parallel, intelligent microenvironment responsive regulatory systems should be developed that autonomously modulate nanozyme catalytic activity in accordance with the real time status of the TME, thereby circumventing both overtreatment and undertreatment and progressively enabling a transition from passive therapy toward active, personalized precision medicine, ultimately achieving closed loop management of the entire therapeutic process. Such a theranostic platform can also be adapted for the combined application of CDT and immunotherapy, enabling simultaneous monitoring and precise regulation of immune response levels, TME changes, and therapeutic efficacy during the combination treatment process, thereby further enhancing the safety and effectiveness of the combination therapy.

Fourth, to elucidate the molecular mechanisms underlying the currently incompletely understood interactions between CeNZs and the TME, and to bridge the gap between the laboratory and the clinic, cutting-edge technologies such as single-cell sequencing, spatial transcriptomics, and organoid models should be employed to deeply dissect the interactions between the nanozymes and tumor cells as well as various components of the TME. Investigations should focus on how CeNZs-mediated CDT modulates the tumor immune microenvironment, elucidate the synergistic mechanisms of CDT combined with immunotherapy, and identify key targets and optimization strategies for the combination therapy. In the medium term, validation in large animal models should be advanced to simulate clinical tumorigenesis and treatment scenarios, rigorously evaluate therapeutic efficacy and safety, and specifically focus on validating CeNZs-mediated CDT combined with immunotherapy regimens in large animal models, thereby providing more reliable experimental evidence for clinical translation. In the long term, the establishment of industry-academia-research collaboration platforms should be promoted to facilitate the scalable production of nanozymes and the formulation of quality control standards, thereby progressively advancing clinical trial applications and translating fundamental research outcomes into clinically feasible oncotherapy strategies. In particular, priority should be given to exploring clinical trials of CeNZs-mediated CDT combined with immunotherapy, fully leveraging the transformative role of immunotherapy in oncology to achieve the dual therapeutic effect of “catalytic killing plus immunological memory”, and opening new avenues for the eradication of malignant tumors. Through the implementation of these phased and well-defined action lines, CeNZs are expected to ultimately emerge as a novel candidate modality in clinical precision oncology, providing an entirely new technological foundation for the treatment of malignant tumors.
