# Supplementary figures and images for: Cerium-based nanozymes for chemodynamic therapy: tumor microenvironment-responsive mechanisms and applications
Source: Front Chem. 2026 May 8;14:1845768. doi: 10.3389/fchem.2026.1845768 (PMC13194537; doi:10.3389/fchem.2026.1845768)

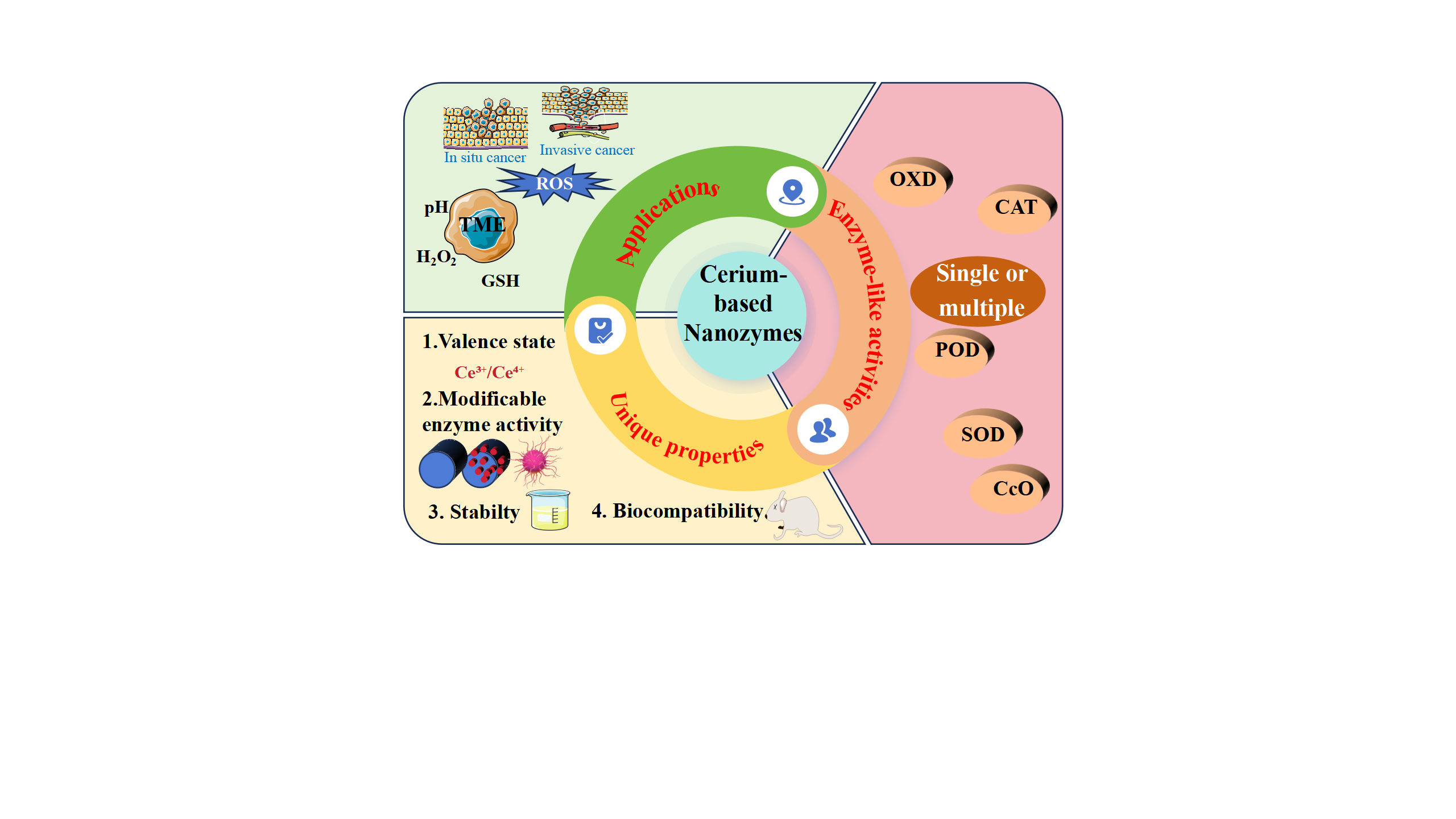

Supplement: Supplementary file 1 [file Image1.png]
